# Supplementary figures and images for: A comparative epigenome analysis of gammaherpesviruses suggests cis-acting sequence features as critical mediators of rapid polycomb recruitment
Source: PLoS Pathog. 2019 Oct 31;15(10):e1007838. doi: 10.1371/journal.ppat.1007838 (PMC6932816; doi:10.1371/journal.ppat.1007838)

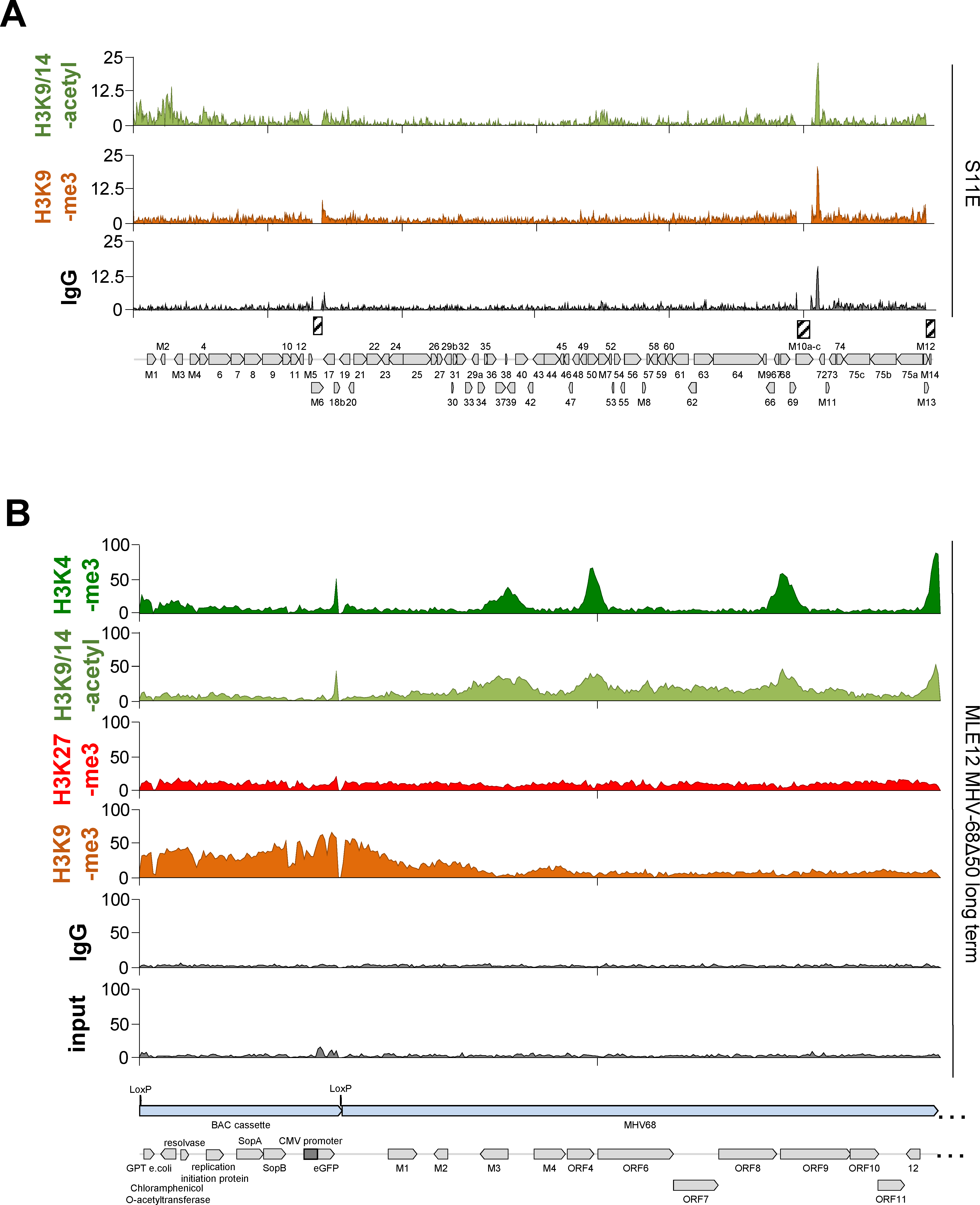

Supplement: S1 Fig — Shown are coverage data from ChIP-seq experiments performed with the indicated antibodies for (A) the MHV-68 genome in S11E cells or (B) the bacmid backbone at the leftmost end of the genome in MHV-68Δ50 BAC-infected MLE12 cells. For the latter, ChIP-seq data from the same samples shown in Fig 3B were mapped to the MHV-68 reference genome including the sequence of the BAC-cassette. For further details please refer to the legends of Figs 1 and 3. (TIF) [file ppat.1007838.s001.tif]

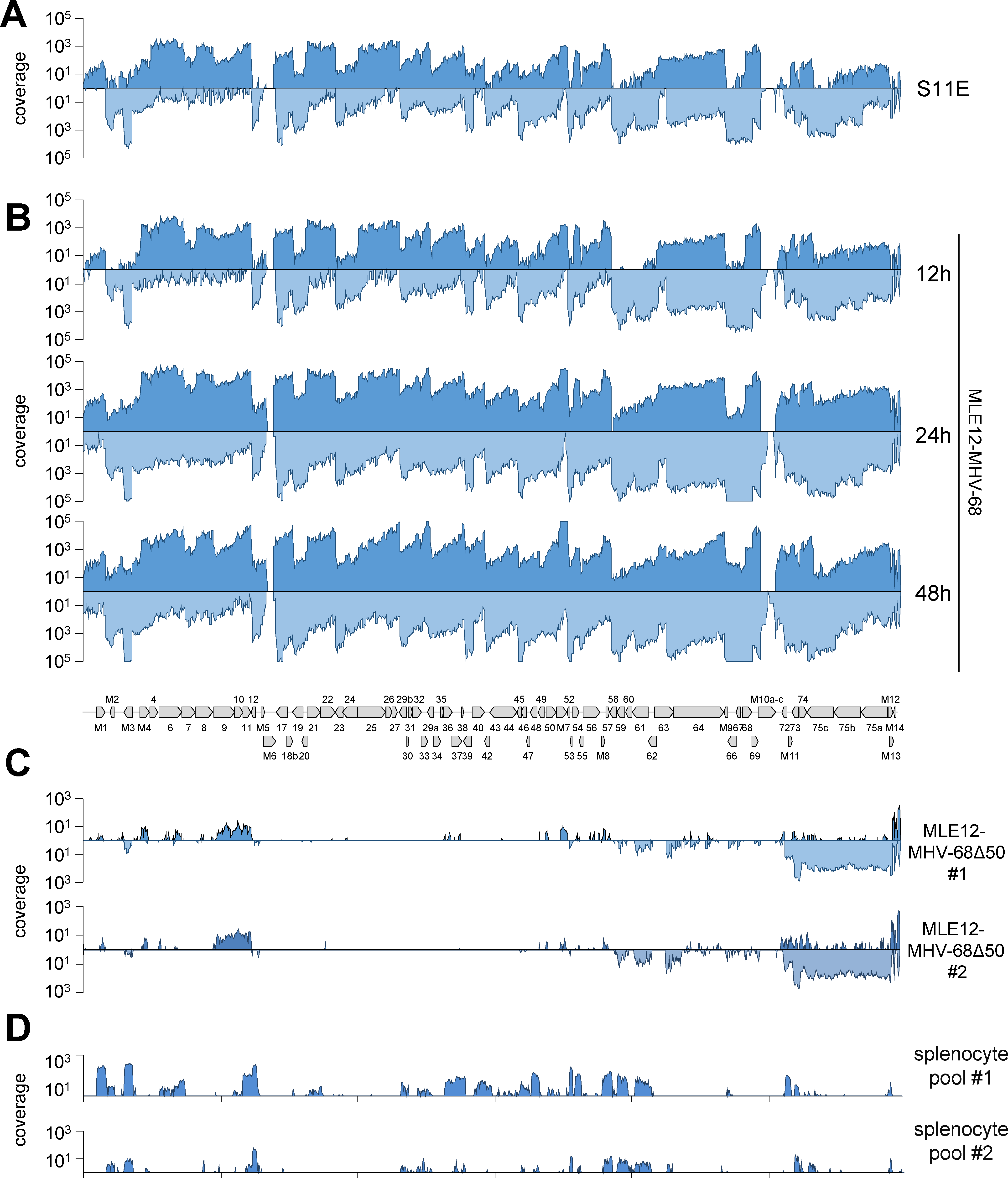

Supplement: S2 Fig — (A) RNA-seq analysis of persistently infected S11E cells (upper panel) or de novo MHV-68 infected MLE12 cells at 12, 24 and 48 hours post infection (lower panels). (B) RNA-seq analysis of two independent GFP-sorted MLE12 cell cultures which had been infected with MHV-68Δ50 for more than 3 weeks. (C) RNA-seq analysis of two splenocyte pools isolated from MHV-68-H2BYFP infected mice (3 mice per pool) at 17 days post infection. RNA sequencing for A and B was performed using a strand-specific sequencing protocol, for C a non-strand-specific, ultra-low input kit was used. Paired-end RNA-seq reads and single reads (for the low cell RNA-seq) were mapped to the MHV-68 reference sequence (NC_001826) using the splice-sensitive STAR pipeline (see Material and methods for details). Coverage tracks depict mean coverage across 100 bp binning windows. For strand-specific data in A and B, forward and reverse strand coverage is shown in the upper and lower plots of each panel. Plots in C show coverage across both strands. (TIF) [file ppat.1007838.s002.tif]

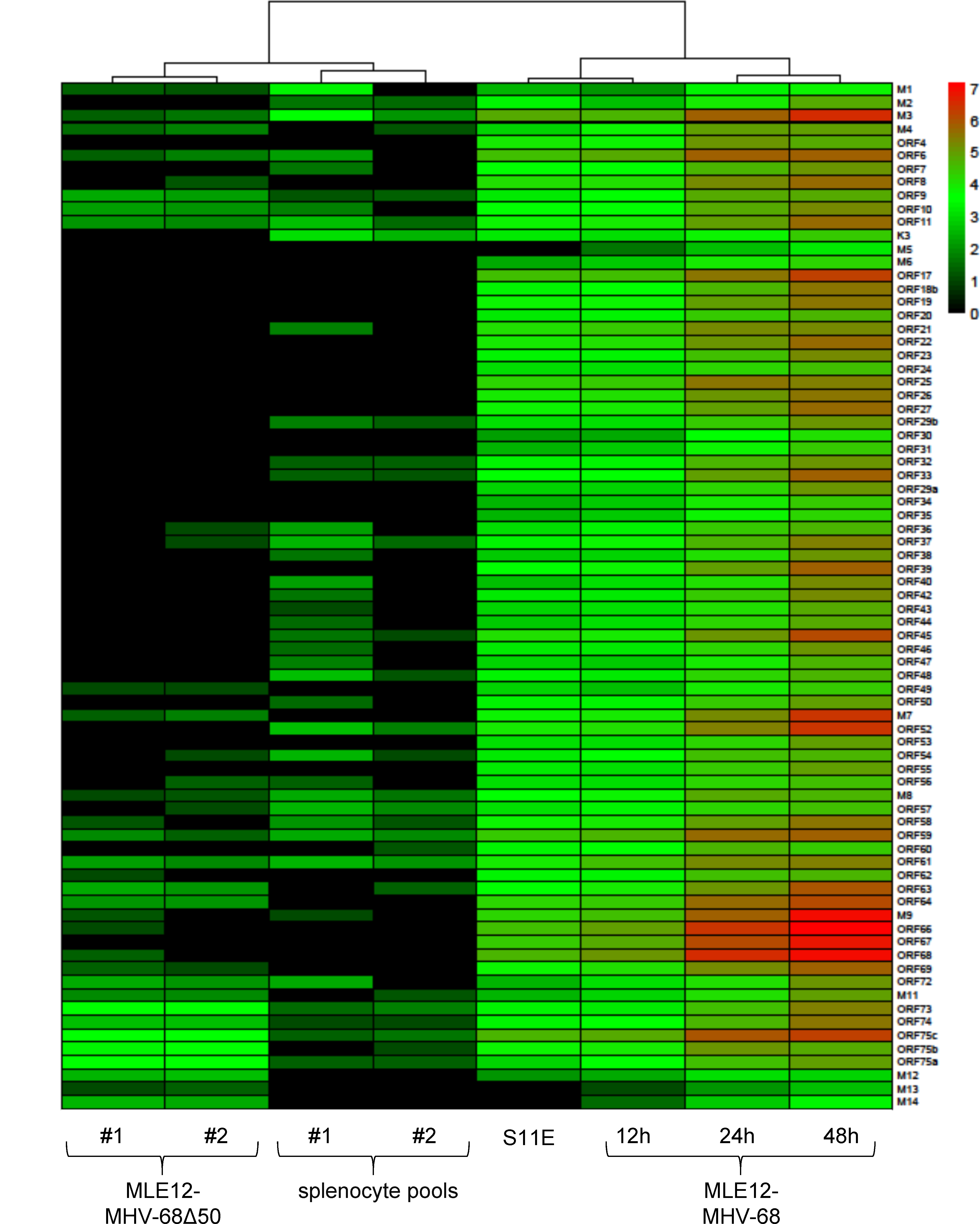

Supplement: S3 Fig — Heatmaps and hierarchal clustering (see tree at top) of normalized feature counts across individual MHV-68 ORFs annotated in the NC_001826 GenBank entry for the experiments shown in S2 Fig. (TIF) [file ppat.1007838.s003.tif]

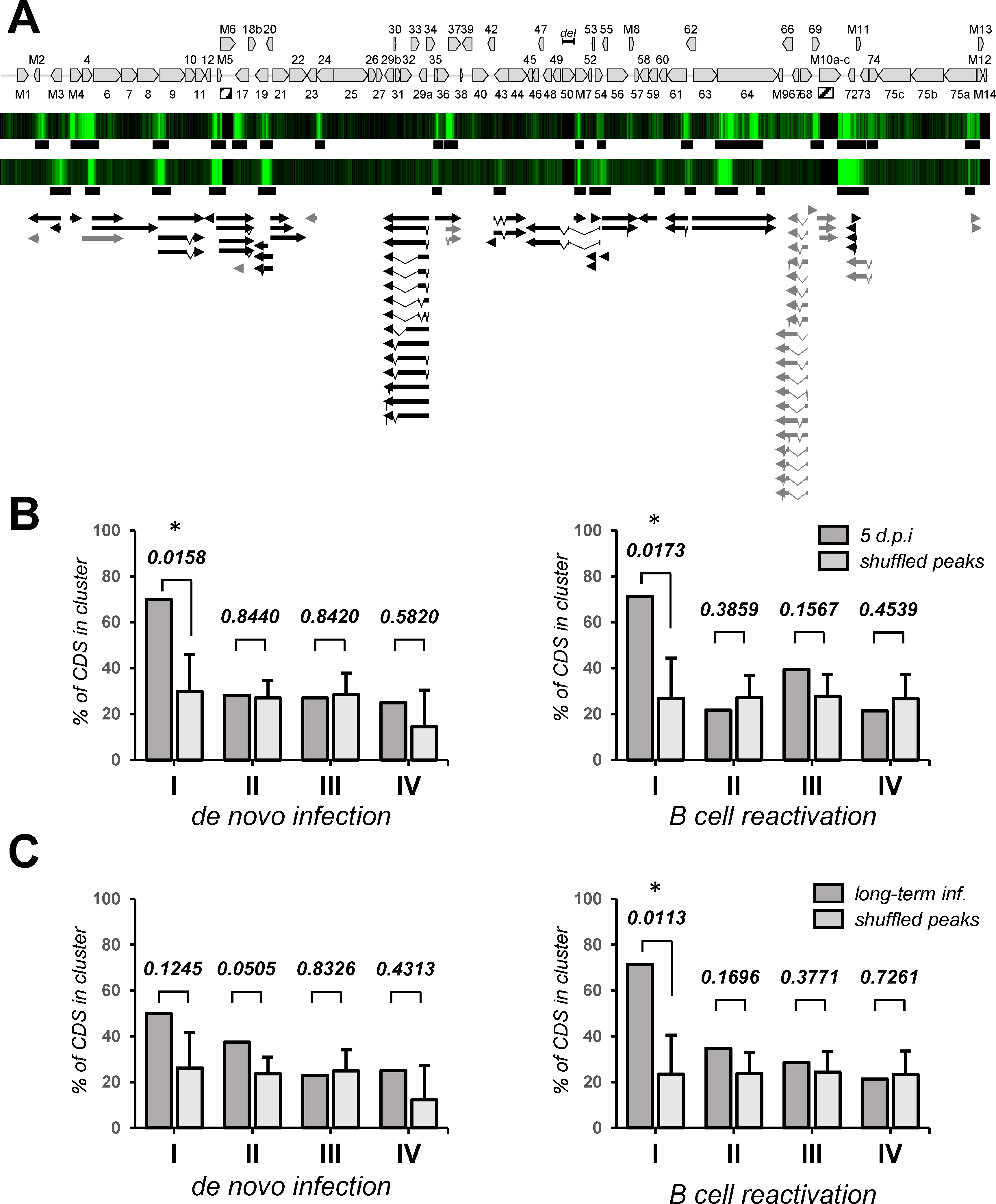

Supplement: S4 Fig — (A) Black and dark grey arrows depict the predicted coding transcripts located downstream of an H3K4-me3 peak (as observed in MHV-68Δ50 infected MLE-12 cells) within a maximum distance of 250bp of their TSS. Transcripts downstream of peaks that are detected at 5 dpi but not in long-term infection are shown in gray. Tracks above transcripts reproduce the H3K4-me3 coverage from Fig 3 (top and bottom track correspond to data from 5 days p.i. or long-term infected cultures, respectively) as a heat map, including the location of peaks detected by MACS14 (indicated by black bars underneath the tracks). (B, C) For each of the 4 expression kinetics clusters (I-IV) defined by Cheng and colleagues [57] for de novo infected fibroblasts (left graphs in each panel) or reactivated B-cells (right graphs) we calculated the percentage of ORFs encoded by transcripts located downstream of H3K4-me3 peaks observed after (B) 5 days of infection or in (C) long-term infected MLE-12 cells (dark grey columns in each graph). Light grey columns and associated error bars represent mean values and standard deviations of analyses repeated 100,000 times with randomly shuffled peaks. Cases with significant (< = 0.05) p-values for the hypothesis that the number of ORFs observed with authentic peaks was significantly above that expected by chance (see S1 Protocol for further details) are indicated. (TIF) [file ppat.1007838.s004.tif]

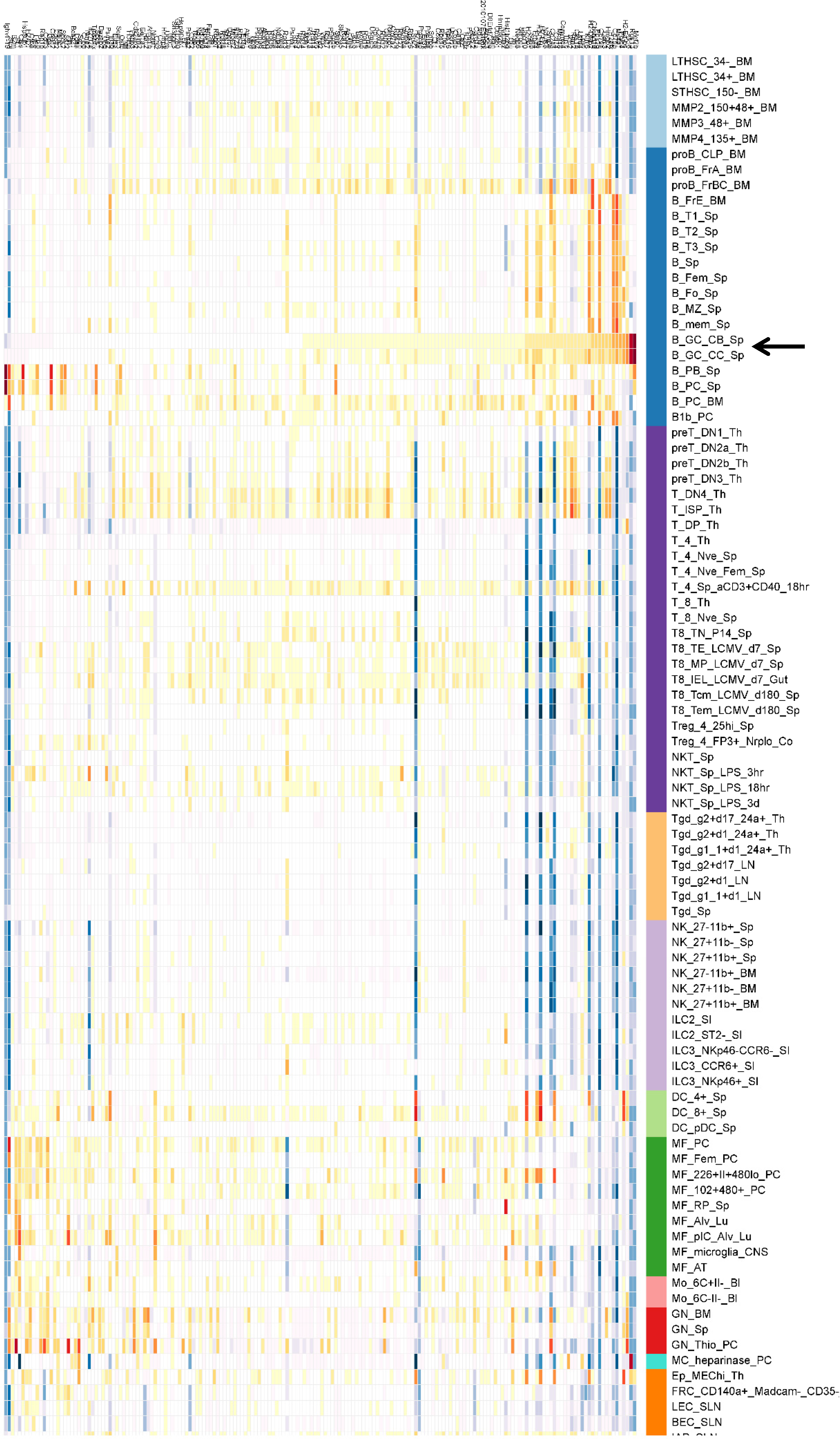

Supplement: S5 Fig — Immgen GeneSet analysis (http://www.immgen.org) of the top 200 expressed genes (as judged by STAR transcriptome analysis) from ultra-low input RNA-seq data of 1000 pooled splenocytes isolated from mice infected with MHV-68-H2BYFP 17 days post infection (see Results and Material & methods sections for details). The heatmap indicates the RNA-seq based row mean normalized expression values of the respective gene ID list for all immune cells within the Immgen database. Germinal center B-cells are indicated with an arrow. (TIF) [file ppat.1007838.s005.tif]

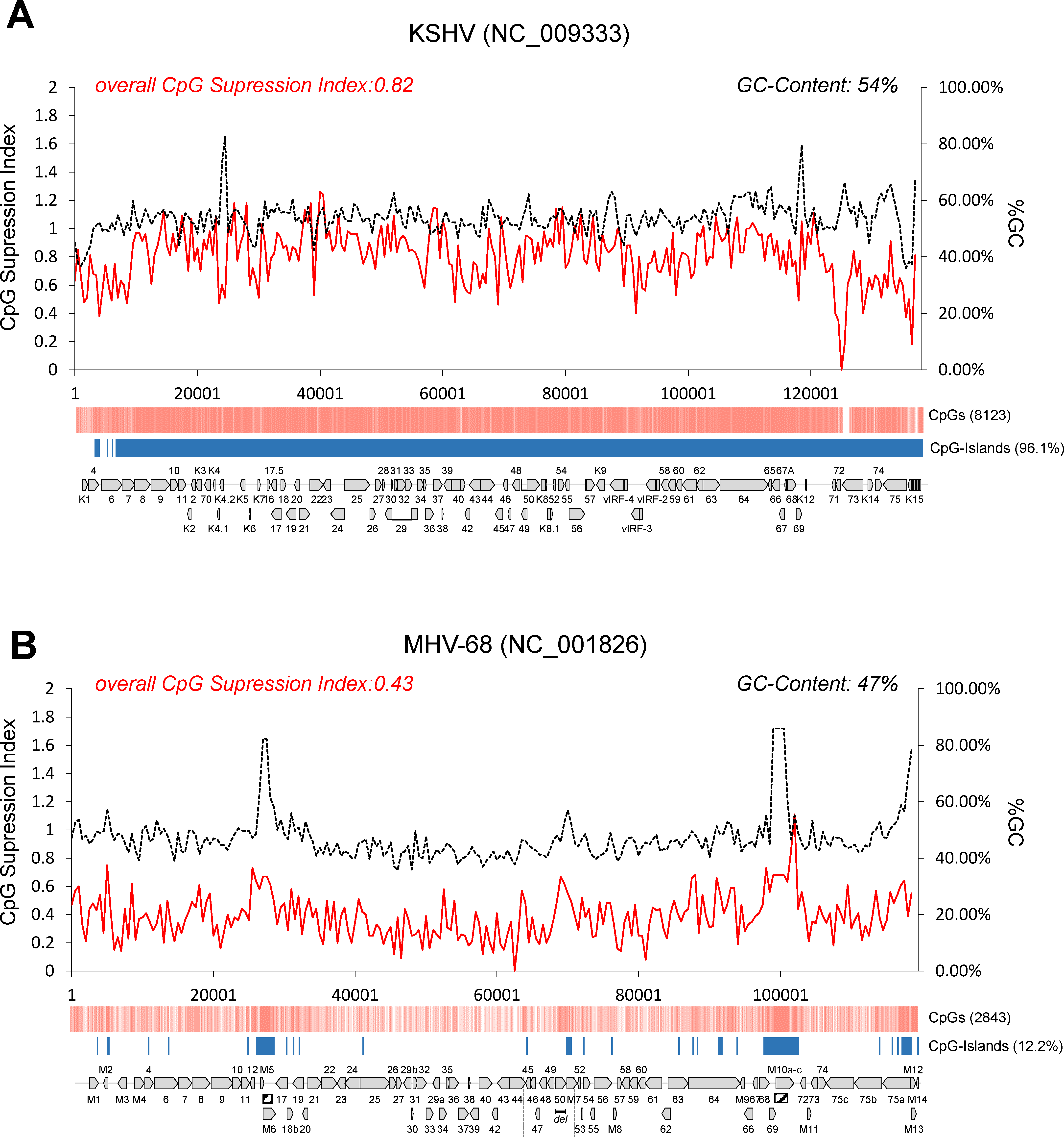

Supplement: S6 Fig — Graphs show GC content (black dashed line, right y-axis) and CpG supression index (red solid line, left y-axis) in a window of 500bp shifted in 250bp steps across the RefSeq genome sequences of (A) KSHV (GenBank accession NC_009333) or (B) MHV-68 (GenBank accession NC_001826). Overall CpG supression index and GC-content is indicated above the graph in each panel. The distribution of CpG motifs is shown in a map underneath the graphs, where the position of each individual motif is indicated by a vertical light-red line. The total number of CpG motifs is given to the right of the map. Blue bars below the CpG map indicate regions which register as CpG islands when employing the same criteria commonly used to designate host cell CpG islands (length > = 200bp, GC-content > = 50%, CpG suppression index > = 0.6). CpG islands were predicted by shifting a 200bp window in steps of 100bp across the viral genomes. Adjacent positive windows were then iteratively joined as long as the qualification criteria were satisfied by the merged region. The overall percentage of the viral genome that qualifies as a CpG Island is given to the right. (TIF) [file ppat.1007838.s006.tif]

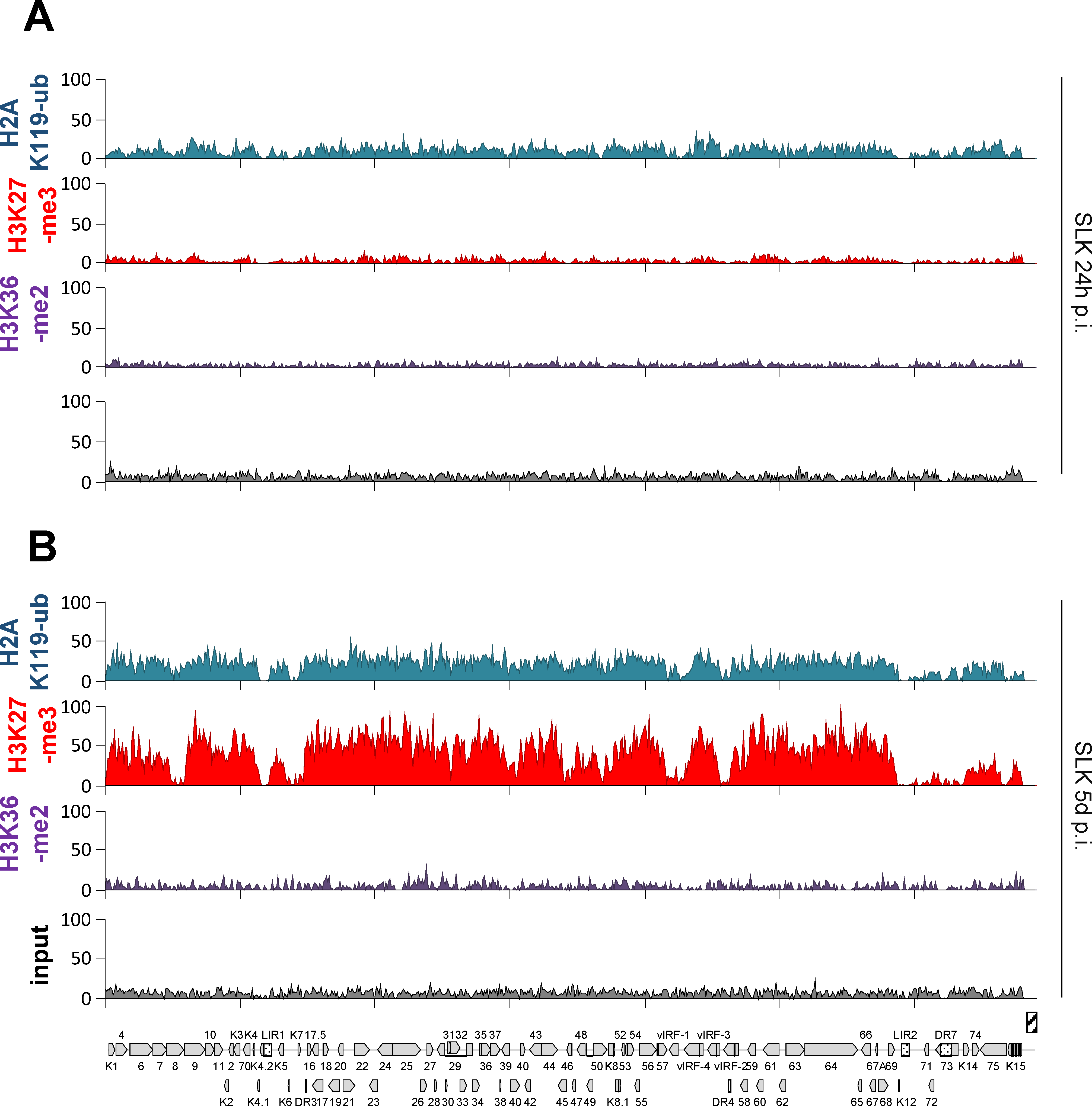

Supplement: S7 Fig — ChIP-seq coverage across the KSHV genome for (top) H2AK119-ub, (2nd from top) H3K27-m3, (2nd from bottom) or (bottom in each panel) input in SLK cells 24 hours (A) or 5 days (B) after infection with KSHV. (TIF) [file ppat.1007838.s007.tif]

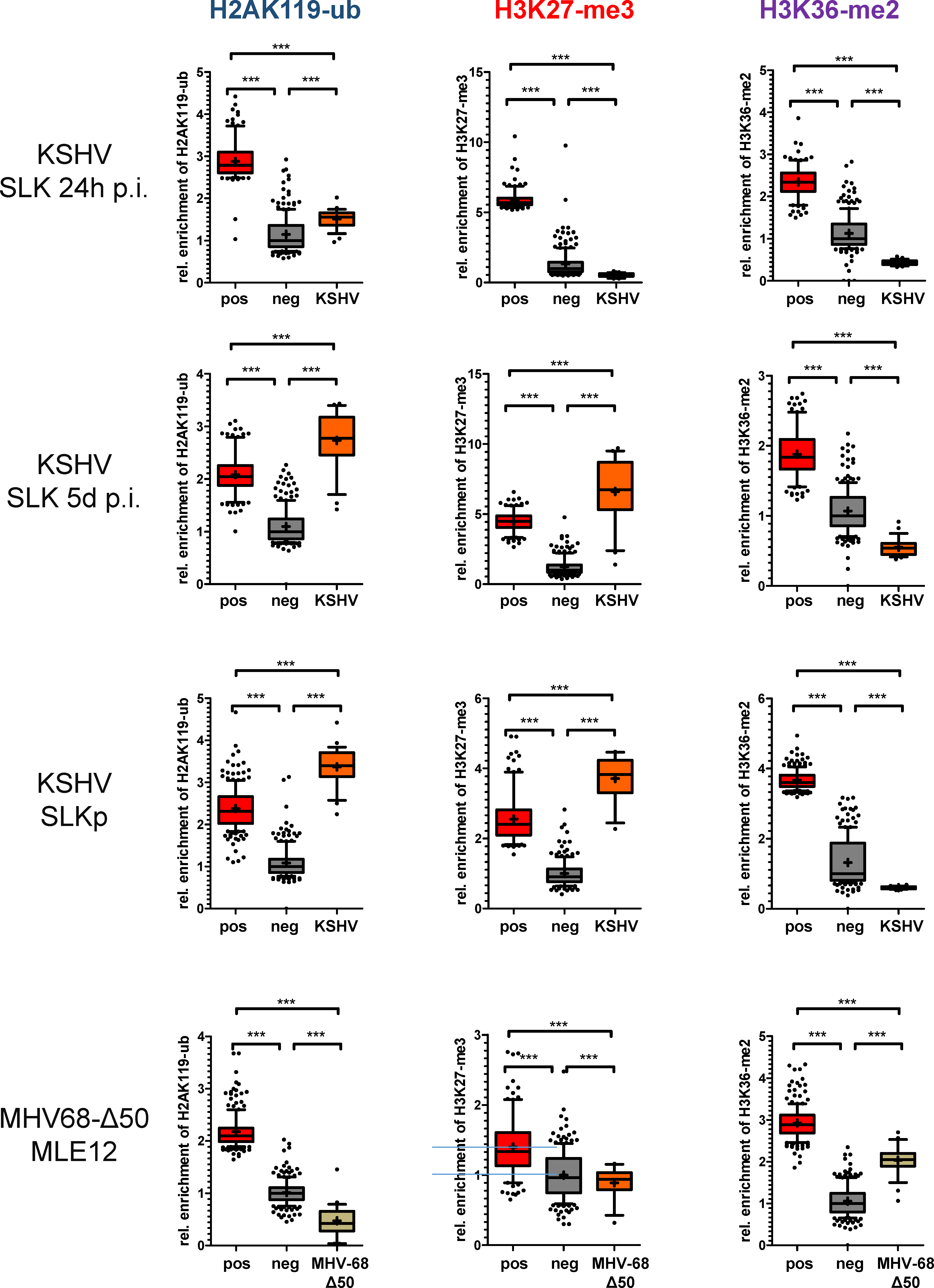

Supplement: S8 Fig — Enrichment of H2AK119-ub, H3K27-me3 and H3K36-me2 was analyzed using the statistical method described in the legend to Fig 4 and the materials and methods section. Data from MLE12 and SLKp cells correspond to those shown in Fig 9E and 9F for H2AK119-ub and H3K36-me2, or those in Fig 4 for H3K27-me3. Coverage tracks for SLK cells at 24 h.p.i and 5 d.p.i. are provided in S7 Fig. (TIF) [file ppat.1007838.s008.tif]

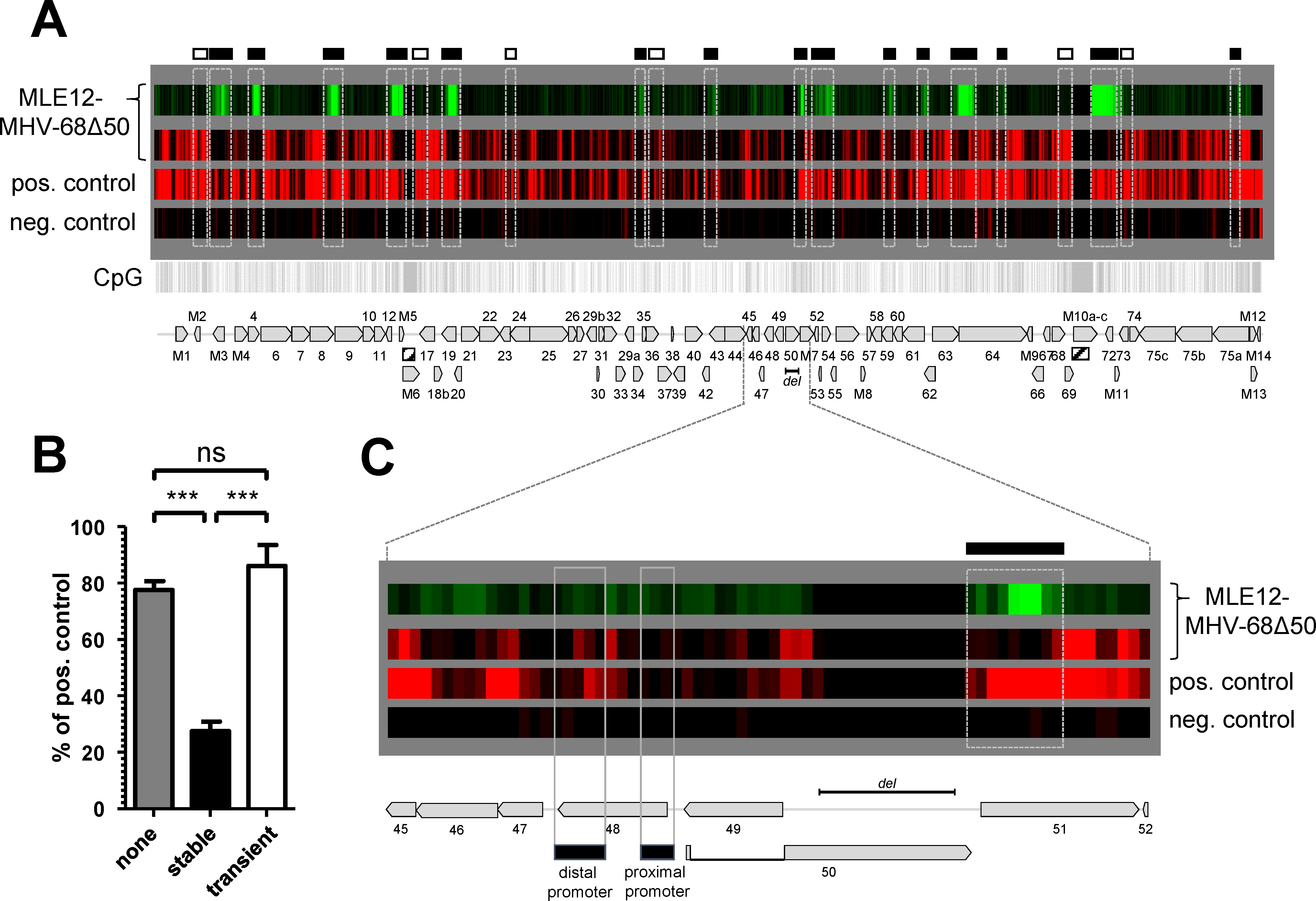

Supplement: S9 Fig — DNA methylation levels were detected by MeDIP-seq using highly pure genomic DNA extracted from long-term MHV-68Δ50-infected MLE12 cells. We generated a positive control sample by in vitro methylation of MHV-68-BAC DNA that was spiked into genomic DNA from MLE12 cells to reflect authentic episome copy numbers in infected cells, as estimated by qPCR. Likewise, we used MLE12 DNA supplemented with unmethylated MHV-68-BAC DNA as negative control. Read coverage was normalized by total mapped read counts and viral input DNA to generate directly comparable tracks. (A) Episome-wide MeDIP-seq analysis. The three lower heat map tracks (in red) indicate relative MeDIP-seq coverage (normalized to the positive control) in the individual samples. The upper track (in green) reproduces the H3K4-me3 coverage data from Fig 3B. Boxes at the top of the panel indicate H3K4-me3 peaks detected by MACS in MHV-68Δ50-infected MLE12 cells. Filled boxes represent stable peaks which persist in long-term infected cells, whereas open boxes indicate transient peaks which are only observed at 5 days post infection. The peak positions are furthermore indicated by dashed frames overlaying the heat map panels. Hashed boxes in the genome map at the bottom indicate repetitive regions unsuitable for analysis and the dotted box marks the ORF50 deletion. (B) Quantification of relative DNA methylation levels (in percent of the positive control) from A in regions which do not acquire any H3K4-me3 peaks (none), or in stable or transient peaks (see legend to panel A for further information). Only persistent peaks protect viral DNA from methylation in long-term infected cells. Data are presented as mean ± SEM. (C) Detailed view of the ORF50 promoter region. The proximal and distal ORF50 promoters are marked by rectangles. The dotted box marks the deletion in the ORF50 coding region. (TIF) [file ppat.1007838.s009.tif]

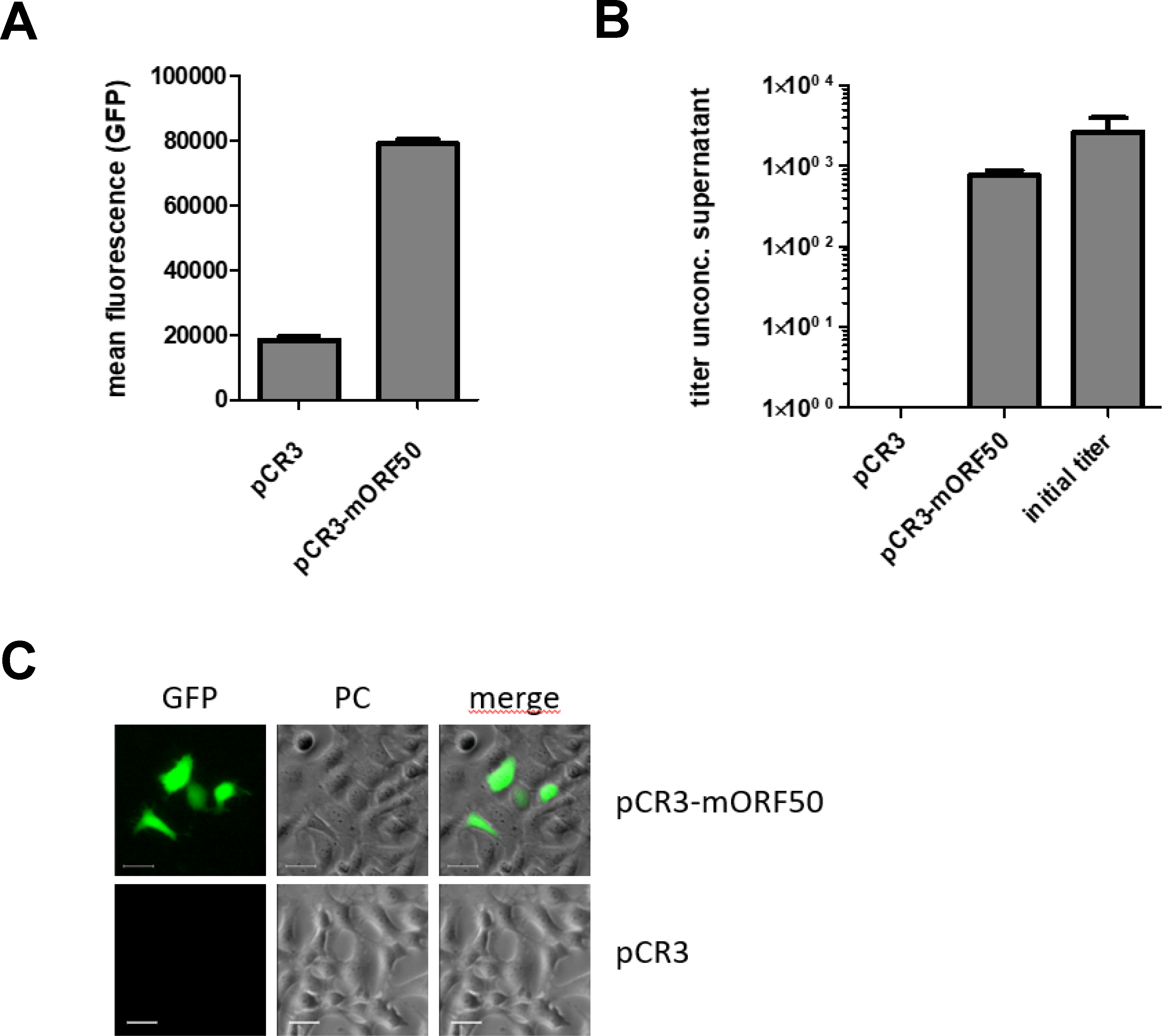

Supplement: S10 Fig — (A) Ectopic expression of MHV-68 ORF50 (mORF50) results in increased GFP expression from MHV-68Δ50 genomes in long-term infected MLE12 cells. MLE12 cells that had been infected with MHV-68Δ50 for more than 3 weeks were transfected with empty vector (pCR3) or a mORF50 expression construct (pCR3-mORF50) to induce lytic reactivation. FACS analysis at 3 d.p.i. demonstrates increased mean GFP fluorescence in pcr3-mORF50 transfected cultures, indicating increased gene expression and viral DNA replication. (B) Long-term MHV-68Δ50 infected MLE12 cells produce infectious virus upon ectopic expression of mORF50. Left and center columns show titers of supernatants collected from long-term infected MLE12:MHV-68Δ50 cells transfected with pCR3 or pCR3-mORF50, respectively. The initial titer of the supernatants that were used to infect MLE12 cultures (produced by transfection of stably mORF50-expressing BHK-21 cells with BAC-MHV-68Δ50, see Methods section for details) is shown in the right column for comparison. Viral titers were determined by infection of fresh MLE12 cultures with unconcentrated, filtrated supernatants. Infectious virus was only produced by pCR3-mORF50 transfected MLE12:MHV-68Δ50 cells, and titers were comparable to the initial titer of MHV-68Δ50 virus harvested from mORF50 expressing BHK-21 cells (n> = 2, mean ± SEM). (C) Fluorescence microscopy images show the presence of newly infected, GFP-positive MLE12 cells 5 days after inoculation with supernatants from pCR3-mORF50 (top), but not pCR3 transfected long-term infected MHV-68Δ50 cells (scale bar = 25 μm, PC: phase contrast). (TIF) [file ppat.1007838.s010.tif]
