# Supplementary material for: A comparative epigenome analysis of gammaherpesviruses suggests cis-acting sequence features as critical mediators of rapid polycomb recruitment
Source: PLoS Pathog. 2019 Oct 31;15(10):e1007838. doi: 10.1371/journal.ppat.1007838 (PMC6932816; doi:10.1371/journal.ppat.1007838)
Supplement: S1 Protocol — (PDF) [file ppat.1007838.s016.pdf]

Functional annotation of activation-associated histone marks in latently replicating MHV-68Δ50-genomes suggests that early lytic genes are pre-marked for rapid expression

To elucidate whether activation-associated histone marks are preferentially found at promoters of specific classes of viral genes we analyzed the location of H3K4me3 peaks relative to the position of transcriptional start sites. In particular, we aimed at investigating whether such genes may potentially share similar expression patterns in a primary lytic infection, or upon reactivation from latency. However, although previous microarray studies have provided detailed data regarding the expression kinetics of individual MHV-68 ORFs [1, 2], the location of putative promoters and transcriptional start sites, as well as the overall coding capacity and splicing structure of the viral transcriptome have thus far not been comprehensively analyzed. The generation of such datasets is greatly complicated by the fact that herpesviral transcription units have a much more complex structure than their cellular counterparts, including extremely dense packaging of coding regions, frequent use of alternative start sites and polyA-site skipping. As a result of these unusual features, algorithms designed for unsupervised mRNA prediction of cellular genes usually fail to accurately annotate viral transcripts. Using our RNA-seq datasets from cells undergoing productive MHV-68 infection, we therefore applied a custom analysis pipeline [3] to identify major transcriptional units and splice products encoding individual ORFs. The methodological approach of our transcriptome and correlative ChIP-seq analysis is described below, and the results of the analysis are depicted in S3 Fig. Information about detected poly-A sites, splice junctions and frequencies, as well as predicted primary and processed transcripts is provided in S5 Dataset. For the primary and processed transcripts, the dataset also provides information regarding putative coding potential and relative abundance in individual samples.

1. Computational Analysis of Viral Transcription Units

Detection of **polyA sites** was performed by searching our stand-specific paired-end RNA-seq data for second-in-pair reads containing a non-genomic polyT tract fused to MHV-68 sequences. For this purpose, we filtered second-in-pair reads from all RNA-seq experiments (primary read data are

available via the European Nucleotide Archive (ENA), accession number PRJEB19354) and selected all sequences beginning with a stretch of at least five thymidines. Reads where then aligned to the MHV-68 reference genome (NC_001826). Alignments were filtered to contain at least two mismatches within the last five bases of the putative polyA tail sequences with regard to the genomic reference, to exclude alignments of reads starting at genomic homopolymeric stretches. Furthermore alignments beneath a cutoff mapping quality of 10 were discarded to exclude putative low quality based misalignments. The base immediately upstream of polyA tail was designated as putative polyA site. Sites were counted and merged using BEDTools v2.19.1 [4] with the count (mode) option and a binning size of 10 bp. This step reports the most abundant position within a dense cluster of putative polyA sites. The score corresponds to the accumulated number of aligned reads passing our filtering criteria. Sites with a score of less than two aligned reads were discarded. Additionally, each site was tested for the presence of canonical polyA signals (AAUAAA and AUUAAA) within 50 bp upstream of the tested site. A list of all identified polyA sites is given in S5 Dataset (sheet 'polyA sites').

Splice junctions within viral transcripts were detected by STAR by analyzing pooled read data from all RNA-seq experiments to generate a single list of putative MHV-68 splices. Subsequently, we binned the data by donor positions and investigated all datasets separately by counting all reads mapping to individual splice junctions, as well as reads traversing the unspliced donors with at least 15 bases on either side of the junction or the unspliced donor respectively allowing for a maximum of two mismatches The fraction of reads mapping to a given splice junction (relative to the total number of reads traversing the donor position in spliced or unspliced form) was calculated to provide a measure of the frequency with which a junction was observed at a given donor site in each sample. To exclude spurious events and potential false positives from downstream analyses, we filtered the data for donors with a total of at least 20 reads encompassing the donor position and a minimum of 2 independent spliced reads accumulated across all samples. A list of all filtered junctions along with read counts and frequencies observed in the individual samples is provided in S5 Dataset (sheet 'splice junctions').

Viral transcripts were predicted using a custom pipeline, given that automated mRNA prediction/assembly algorithms (e.g. TopHat/Cufflinks, Trinity) failed due to the high density of transcriptional units in combination with abundant antisense transcription. To predict **primary transcipts**, we first identified the position of likely transcriptional start sites (TSS) by inspecting strand-specific mapping data from all RNA-seq experiments for regions of substantial coverage increase, annotating a putative TSS immediately upstream of the coverage slope. Putative primary transcripts were then predicted by evaluating TSS data in combination with coverage data and the position of experimentally identified polyA-sites (see above). All predicted primary transcripts are listed in S5 Dataset (sheet 'primary transcripts'). In rare cases (marked in S5 Dataset), a significant drop of read coverage between an individual TSS and an adjacent transcriptional unit was observed but no polyA-site had been detected. This may occur if the 3'end of transcripts is poorly covered (e.g., due to structural properties). In these cases, similar to the identification of TSS, we inferred the approximate position of the 3-'end of the primary transcript from the coverage data, annotating the position immediately downstream of the downward coverage slope. The structure and relative abundance of **processed mRNAs** were predicted by incorporating the junction data (see above) as described previously [3]. Briefly, for each primary transcript, we considered the contingent of mRNAs containing all possible combinations of junctions that mapped within the bounds of the transcriptional unit. The frequency with which an individual junction was detected in a given sample can be reasonably assumed to represent the probability with which it occurs in its parental primary transcript. For each spliced transcript, we therefore calculated the combinatorial product of individual junction frequencies to provide an estimate of its abundance relative to the primary transcript. To exclude transcripts that would be expected to be exceedingly rare we filtered all predicted mRNAs for transcripts with an estimated relative frequency of at least 0.001 (corresponding to 0.1% of the parental primary transcript) in at least one of the analyzed samples. The 650 predicted transcripts which passed this filter are listed in sheet 'processed transcripts' of S5 Dataset, together with their estimated relative abundance in individual samples. The table additionally indicates whether or not the predicted TSS of

a given mRNA is located within a distance of 250 bp of an H3K4me3 peak in any of the samples analyzed by ChIP-seq.

To evaluate the potential of processed mRNAs to express ORFs annotated in the RefSeq GenBank entry NC_001826, we detected all annotated ORFs that were fully contained within each transcript and designated the 5'-proximal ORF as a putative translation product. Starting from the 5'-end, the putative 5'-UTR was then scanned for consecutive non-annotated open reading frames with a mimimal length of 10 codons. To designate a given mRNA as a putative coding transcript for the annotated 5'-proximal ORF, we allowed a maximum number of 5 short uORFs (length <= 30 codons) and additionally a single longer upstream ORF with a start codon in a non-Kozak context. The results of this analysis are shown in the datasheet 'processed transcripts' of S5 Dataset.

2. Correlative Analysis of H3K4-me3 Peaks

We used our curated dataset to generate a list of all viral ORFs that can be potentially expressed from viral regions enriched for H3K4-me3 in MHV-68Δ50-infected cells, and correlated this information with data from a recent tiling microarray study [1] that had determined viral expression kinetics of *de novo* infected fibroblasts in comparison to reactivating B-cells, assigning viral ORFs to one of four temporal expression clusters (I through IV) in each mode of productive infection. Based on this elegant dataset, we matched the viral ORFs encoded by transcripts with a transcriptional start site in a distance of maximally 250bp of a H3K4-me3 peak (see S3 Fig A, S4 Dataset for peak locations) to the clusters defined by Cheng et al. We then calculated the percentage of ORFs with associated H3K4-me3 peaks at 5dpi (S3 Fig B) or in long-term infected cells (S3 Fig C) for each cluster from *de novo* infected fibroblasts or reactivated B cells (left and right panels, respectively, in S3 Figs B and –C; see also table at the end of this document for information on individual ORFs). To evaluate whether an overrepresentation within a given cluster in our experimental dataset was significant, we performed the same analysis 100,000 times *in silico* with randomly shuffled H3K4-me3 peak regions. The data shown in S3 Figs B and -C under 'shuffled peaks' (light grey bars) represent mean and standard deviation of counts across all shuffling experiments, whereas p-values represent the relative fraction

of shuffling experiments (i.e., the number of experiments divided by 100,000) in which the counts of positive ORFs was equal to or greater than the counts obtained with the authentic peak distribution (dark gray bars in S3 Figs B and -C).

As shown in S3 Fig B, this association analysis revealed that, at five days post infection with MHV-68Δ50, 70% of all ORFs classified as cluster I *de novo* infected fibroblasts as well as reactivated B-cells were associated with H3K4-me3 peaks. This represents a significant enrichment compared to our shuffling experiments, where only 25-30% of cluster I ORFs were associated with a peak. Interestingly, during long-term infection there is only a significant enrichment of H3K4-me3 associated ORFs with cluster I genes from reactivated B cells but not the *de novo* infected fibroblasts (S3 Fig C). Hence, our results suggest H3K4-me peaks acquired by a strictly latent MHV-68 mutant at day 5 post-infection preferentially occupy genes expressed immediately after a productive infection, whereas those that persist during long term infection are predominantly associated with genes that are expressed with immediate early kinetics upon reactivation from latency.

Table: Correlation of H3K4-me3 peaks with TSS' of putative coding transcripts.

ORFs that can be putatively expressed from a predicted TSS that overlaps with an H3K4-me3 peak detected at 5d p.i. or in long term infection are marked with "+". For details of the analysis refer to the Methods section. The columns entitled "kinetics" indicate the expression kinetics cluster for de novo infected fibroblasts (de novo) and reactivated B-cells (react) as determined and published previously by Cheng et al. (*nd* not detected, *na* not available).

	H3K4-me3 at TSS		kinetics (Cheng et al.)				H3K4-me3 at TSS		kinetics (Cheng et al.)	
ORF	5d p.i.	long-term	de novo	react.		ORF	5d p.i.	long-term	de novo	react.
M1			IV	II		ORF43			III	III
M2	+		II	III		ORF44		+	I	II
M3		+	II	II		ORF45			II	III
M4	+	+	II	II		ORF46			II	III
ORF4	+		III	IV		ORF47			II	IV
ORF6	+	+	II	III		ORF48			II	II
ORF7			IV	nd		ORF49	+	+	II	III
ORF8			III	III		ORF50			I	II
ORF9	+	+	II	I		M7	+	+	IV	IV
ORF10			II	I		ORF52		+	II	III
ORF11			II	II		ORF53		+	III	III
ORF12	+	+	II	II		ORF54	+	+	I	I
M5[a]	+	+	na	na		ORF55		+	III	III
M6[a]	+	+	na	na		ORF56	+	+	I	III
ORF17			III	III		M8			na	na
ORF17.5	+		III	III		ORF57			I	II
ORF18b			II	III		ORF58			II	II
ORF19	+	+	III	III		ORF59		+	II	II

ORF20		+	III	II		ORF60			II	II
ORF21	+	+	II	II		ORF61	+	+	I	I
ORF22			II	II		ORF62			III	III
ORF23	+		III	III		ORF63		+	III	IV
ORF24			II	II		ORF64			IV	IV
ORF25			III	IV		M9	+		III	IV
ORF26			III	III		ORF66			III	III
ORF27			III	III		ORF67	+		III	III
ORF28			III	III		ORF67a			III	IV
ORF29b[a]		+	na	na		ORF68			III	IV
ORF29a/b		+	III	IV		ORF69	+		III	III
ORF30			II	II		M10a[a]	+		na	na
ORF31			II	I		M10b[a]	+		na	na
ORF32			II	III		M10c[a]	+		na	na
ORF33			III	IV		ORF72	+	+	II	I
ORF29a[a]		+	na	na		M11	+	+	II	I
ORF34			II	II		ORF73	+		I	III
ORF35			II	III		ORF74			II	II
ORF36	+	+	I	III		ORF75c			III	IV
ORF37	+		I	II		ORF75b			II	IV
ORF38	+		I	II		ORF75a			nd	IV
ORF39			III	III		M12[a]	+		na	na
ORF40			II	II		M13[a]	+		na	na
ORF42		+	II	II		M14[a]			na	na

[a] ORFs that were not part of the dataset by Cheng et al. were excluded from the analysis presented in Fig 4.

References

1. Cheng BY, Zhi J, Santana A, Khan S, Salinas E, Forrest JC, et al. Tiled microarray identification of novel viral transcript structures and distinct transcriptional profiles during two modes of productive murine gammaherpesvirus 68 infection. J Virol. 2012;86(8):4340-57. Epub 2012/02/10. doi: 10.1128/JVI.05892-11. PubMed PMID: 22318145; PubMed Central PMCID: PMC3318610.
2. Johnson LS, Willert EK, Virgin HW. Redefining the genetics of murine gammaherpesvirus 68 via transcriptome-based annotation. Cell Host Microbe. 2010;7(6):516-26. doi: 10.1016/j.chom.2010.05.005. PubMed PMID: 20542255; PubMed Central PMCID: PMCPMC2900189.
3. Theiss JM, Gunther T, Alawi M, Neumann F, Tessmer U, Fischer N, et al. A Comprehensive Analysis of Replicating Merkel Cell Polyomavirus Genomes Delineates the Viral Transcription Program and Suggests a Role for mcv-miR-M1 in Episomal Persistence. PLoS Pathog. 2015;11(7):e1004974. doi: 10.1371/journal.ppat.1004974. PubMed PMID: 26218535; PubMed Central PMCID: PMCPMC4517807.
4. Quinlan AR, Hall IM. BEDTools: a flexible suite of utilities for comparing genomic features. Bioinformatics. 2010;26(6):841-2. Epub 2010/01/30. doi: 10.1093/bioinformatics/btq033. PubMed PMID: 20110278; PubMed Central PMCID: PMC2832824.
