# Supplementary material for: A comparative epigenome analysis of gammaherpesviruses suggests cis-acting sequence features as critical mediators of rapid polycomb recruitment
Source: PLoS Pathog. 2019 Oct 31;15(10):e1007838. doi: 10.1371/journal.ppat.1007838 (PMC6932816; doi:10.1371/journal.ppat.1007838)
Supplement: S2 Protocol — (PDF) [file ppat.1007838.s017.pdf]

Methylated DNA-immunoprecipitation (MeDIP)

MeDIP analysis was essentially performed as described previously [1]. Briefly; genomic DNA was isolated by proteinase K digestion, phenol chloroform extraction and ethanol precipitation. Contaminating RNA was degraded by RNAseA treatment. For MeDIP analysis, we generated a methylation positive control by *in vitro* methylation of an MHV-68 containing BAC construct using the CpG methyltransferase M.SssI (NEB) as described by the manufacturer's instructions. Unmethylated MHV-68 BAC served as negative control. This control DNA was spiked into MLE-12 cell DNA using a ratio similar to long-term MHV-68Δ50 infected MLE-12 cells as judged by qPCR. DNA was diluted in TE buffer and sheared by sonication using a Bioruptor device (Diagenode) to an average fragment size of 300 bp. 5 µg of sheared DNA were denatured in 500 µl TE buffer at 98°C for 10 min, followed by incubation on ice for 10 min. 50 µl denatured DNA were saved as input sample. IPs were performed by adding 51 µl 10x IP-buffer (100 mM Na-phosphate buffer, pH 7.0, 1.4 mM NaCl, 0.5 % Triton X-100) and 2 µg of a 5´-methylcytidine specific antibody (MAb-5MECYT-100, Diagenode). After 2 hrs incubation on a rotating wheel at 4°C, 50 µl prewashed Dynabeads® M-280 Sheep anti-Mouse IgG (Invitrogen) were added to the DNA-antibody complexes and incubated for additional 2 hrs at 4°. Samples were washed three times for 10 min using 1x IP-buffer. DNA was eluted in 200 µl elution buffer (50 mM Tris-HCl pH 8.0, 10 mM EDTA, 1 % SDS) supplemented with 3µl ProteinaseK (40mg/ml). Samples were incubated shaking at 65°C for 30 min. After addition of 200µl TE, DNA was purified twice by standard phenol-chloroform extraction and ethanol precipitation. Precipitated DNA was collected in 15.5 µl H_2O. Input control DNA was diluted to 400µl using TE and Precipitated similar to the MeDIP samples.

MeDIP-seq library preparation, sequencing and data analysis

Sequencing libraries of MeDIP and respective input samples were generated from 1ng DNA using the DNA SMART ChIP-Seq Kit (TaKaRa, Clontech), which is suitable for library generation from single stranded DNA, according to the manufacturer´s instructions. All MeDIP-seq sequencing libraries were sequenced on a HiSeq 2500 system (Illumina) using single read (1x50) flow cells. MeDIP-seq data was mapped to the reference genomes as described for ChIP-seq in the Material and Methods section.

Additionally, data was normalized to the relative amount of viral episomes per sample as determined by the respective input, which results in directly comparable datasets.

References

1 Günther, T. & Grundhoff, A. The epigenetic landscape of latent Kaposi sarcoma-associated herpesvirus genomes. *PLoS Pathog* **6**, e1000935, doi:10.1371/journal.ppat.1000935 (2010).
